# Exploring the Landscape of Psychosurgery in Low and Middle-Income Countries: A Scoping Review Protocol

**DOI:** 10.1192/j.eurpsy.2024.1469

**Published:** 2024-08-27

**Authors:** S. Murthy, J. Wellington, R. Suvarna

**Affiliations:** ^1^University of Bari Aldo Moro, Bari, Italy; ^2^Bradford Teaching Hospitals NHS Foundation Trust, Bradford; ^3^University of Leeds, Leeds, United Kingdom

## Abstract

**Introduction:**

Psychosurgical procedures gained an infamous reputation during the 20th century with the implementation of the lobotomy as treatment for several psychiatric illnesses. However, modern-day psychosurgery is a flourishing field that provides valid treatment alternatives to neuropsychiatric patients thanks to increasingly accurate and safe stereotactic procedures. As more than 80% of people with mental disorders reside in Low and Middle Income Countries (LMICs), investigating the impact of psychosurgical procedures has a global relevance. People living in LMICs are exposed to a variety of stressors which could facilitate the development of psychiatric and neurological diseases. The immense gap that still exists between the population of LMICs and adequate medical and surgical care is an important obstacle to the reduction of global mental health burden. A scoping review will be conducted to investigate the extent of the existing literature and identify key themes, challenges and research gaps on the implementation and outcomes of psychosurgery in LMIC settings.

**Objectives:**

- **To comprehensively map the existing literature:** Provide an extensive overview of the literature on the use of psychosurgery in low and middle-income countries.
- **To identify key themes:** Recognize recurring themes and topics within the literature related to psychosurgery in these settings.
- **To assess challenges:** Analyze the challenges and barriers associated with the implementation of psychosurgery in resource-constrained contexts.
- **To identify research gaps:** Highlight areas within the existing literature where further research is needed to enhance our understanding of psychosurgery in low and middle-income countries.

**Methods:**

The methodology consists of five stages, consistent with Arksey and O’Malley’s framework. Using the PICO model, the Research Question, Inclusion/Exclusion Criteria and search methods were developed. Electronic Medical Databases (Medline OVID, Cochrane Library, Embase, PubMed, Scopus) will be searched for relevant studies. The PRISMA-ScR framework is used to guide the reporting process. Quantitative and Qualitative data will be extracted, including key information such as study type, demographics and methods used to assess the outcomes of psychosurgical interventions. Data will be presented discursively, supported with statistics and graphs where appropriate. No ethical approval is required.

**Results:**

/

**Conclusions:**

The results will be useful to healthcare professionals in LMICs involved in neuropsychiatric care, evaluating the current uses of psychosurgery and their potential benefit for the affected population whilst highlighting gaps in knowledge with the aim of propelling further research.

**Disclosure of Interest:**

None Declared

